# Association Between Skeletal Muscle Mass and Severity of Steatosis and Fibrosis in Non-alcoholic Fatty Liver Disease

**DOI:** 10.3389/fnut.2022.883015

**Published:** 2022-04-26

**Authors:** Wen Guo, Xin Zhao, Mengyuan Miao, Xiuru Liang, Xiaona Li, Pei Qin, Jing Lu, Wenfang Zhu, Juan Wu, Chen Zhu, Nianzhen Xu, Qun Zhang

**Affiliations:** Department of Health Promotion Center, The First Affiliated Hospital with Nanjing Medical University, Nanjing, China

**Keywords:** skeletal muscle mass, hepatic steatosis, liver fibrosis, non-alcoholic fatty liver disease, skeletal muscle mass index

## Abstract

**Background:**

Sarcopenia is known to be the risk factor of non-alcoholic fatty liver disease (NAFLD). However, studies evaluating the association of skeletal muscle mass (SMM) with liver fibrosis by transient elastography are limited. Here, we investigated the association of SMM with hepatic steatosis and fibrosis assessed in Chinese adults.

**Methods:**

Patients who underwent liver ultrasonography at the Health Promotion Center of the First Affiliated Hospital of Nanjing Medical University between January 2020 to June 2021 were enrolled. We used transient elastography to evaluate the degree of hepatic fat and liver stiffness. Appendicular skeletal muscle mass was determined by bioelectrical impedance and was adjusted for body weight to derive the skeletal muscle mass index (SMI).

**Results:**

Of 3,602 finally enrolled individuals, 1,830 had NAFLD and 1,772 did not have NAFLD. SMI gradually decreased as the severity of hepatic steatosis increased (40.47 ± 3.94% vs. 39.89 ± 3.57% vs. 39.22 ± 3.46% vs. 37.81 ± 2.84%, *P* < 0.001). Individuals with F3-F4 and F2 liver fibrosis groups had significantly lower SMI than individuals with F0-F1 stages (37.51 ± 3.19% vs. 38.06 ± 3.51% vs. 39.36 ± 3.38%, *P* < 0.001). As the SMI increased, the percentages of subjects with mild and severe NAFLD, and the percentages of subjects in F2 and F3-F4 stage were gradually decreased. SMI was independently associated with the severity of hepatic steatosis and fibrosis by logistic regression analysis. Moreover, decreased SMI was an independent risk factor for NAFLD and fibrosis.

**Conclusion:**

SMI is closely associated with liver fat content and liver fibrosis in Chinese adults with NAFLD.

## Introduction

Non-alcoholic fatty liver disease (NAFLD) is the most common liver disease in China, affecting about 29% of the population (1). NAFLD can range from simple steatosis, which typically has a benign presentation, to non-alcoholic steatohepatitis (NASH), which can progress to cirrhosis or hepatocellular carcinoma. Moreover, compelling evidence suggests that NAFLD is closely associated with numerous chronic diseases, such as cardiovascular disease (CVD), type 2 diabetes mellitus (T2DM), chronic kidney disease (CKD) and colorectal cancer (2–4). However, to date there is no effective drug for treatment of NAFLD. Given the substantial disease burden and risk of progression, understanding the pathobiology, risk factors for development and progressive disease, and early therapies effective for NAFLD is an important research goal.

The primary tissue responsible for glucose uptake and utilization, and therefore a regulator of peripheral insulin resistance (IR), is skeletal muscle. During the past few years, skeletal muscle insulin resistance has been considered as an important contributor to metabolic diseases (5). More recent reports suggest that low skeletal muscle mass (SMM) serves as an important risk factor for IR related diseases such as T2DM and metabolic syndrome (6, 7). IR has a vital role in the development of NAFLD (8). Based on this theory, the research on the relationship between low SMM and NAFLD has become a hot topic in recent years. Assessment of liver histology obtained through biopsy sampling is the gold standard to diagnose NAFLD and liver fibrosis, but the expense and invasiveness of liver biopsy limits its routine clinical use. Therefore, liver biopsy is not suitable for large-scale epidemiological studies. MRI-based techniques are non-invasive gold standard for assessment of liver fat content, which can be performed in large studies (9, 10). But the MRI-based techniques are expensive and not suitable for participants with stent implantation. Transient elastography is another non-invasive approach to evaluate hepatic steatosis and fibrosis that can remove the need for patients to undergo liver biopsy (11). Recent population studies have indicated that low SMM is correlated with the presence of NAFLD, and advanced fibrosis defined by non-invasive biomarkers or liver histology (12, 13). In addition, sarcopenia was associated with increased risk of all-cause, diabetes-related and cancer-related death in patients with NAFLD (14). However, few epidemiological studies in Chinese populations have been done that define NAFLD using transient elastography. In this study, we aimed to determine the association of SMM with the severity of hepatic steatosis and liver fibrosis presence, as defined by non-invasive transient elastography, in Chinese adults.

## Materials and Methods

### Study Population

The adult participants of this study were those who visited the Health Promotion Center of the First Affiliated Hospital of Nanjing Medical University for a health check-up from January 2020 to June 2021. A total of 3,943 participants who underwent liver ultrasonography were enrolled. Each participant underwent a face-to-face interview to complete a questionnaire including drinking, smoking, medical history, and medication use. Individuals were excluded from the study if they had excessive alcohol use (defined as > 140 g/week for men and >70 g/week for women) and had been previously diagnosed other chronic liver diseases including hepatitis, cirrhosis, autoimmune liver disease and drug-induced liver disease. Participants with hypothyroidism, CKD, malignant tumors, or pregnancy were also excluded. 169 participants were not examined by transient elastography, and 143 participants were not examined by bioelectrical impedance analysis. A total of 3,602 participants who underwent liver ultrasonography, transient elastography and bioelectrical impedance analysis were finally included in the study. All participants gave written consent to participate. This study was realized with the guidelines laid down in the Declaration of Helsinki. The study protocol was approved by the Human Research Ethics Committee of the First Affiliated Hospital of Nanjing Medical University (2020-SR-346).

### Anthropometric and Laboratory Measurements

Height, weight, and seated blood pressure were measured by well-trained nurses in accordance with international standards. Body mass index (BMI) was calculated based on the weight and height. After a 12-h overnight fast, early morning venous blood samples of all participants were taken and subsequently analyzed by a central, certified laboratory at the First Affiliated Hospital of Nanjing Medical University. The laboratory evaluation, including fasting plasma glucose (FBG), lipid profiles, blood liver enzymes, and uric acid were determined by the biochemical autoanalyzer (Chemistry Analyzer Au5800, Olympus Corporation, Tokyo, Japan). We used high-performance liquid chromatography to measure glycated hemoglobin A1c (HbA1c) levels of all participants.

### Liver Fibrosis and Severity of Steatosis

Liver stiffness measurements (LSM) were obtained using the FibroTouch FT100 (Wuxi Hisky Medical Tech. Co., Ltd., Xinwu, Wuxi, China). Severity of hepatic steatosis was determined using the fat attenuation parameter (FAP) on the FibroTouch FT100 device as previously described (15). Participants with NAFLD were grouped by severity according to the FAP value as follows: mild, 240 dB/m ≤ FAP < 265 dB/m; moderate, 265 dB/m ≤ FAP < 295 dB/m; and severe, FAP ≥ 295 dB/m. NAFLD participants with LSM values > 7.3 kPa were classified as having liver fibrosis, and those with LSM ≤ 7.3 kPa were classified as not having liver fibrosis.

### Body Composition and Skeletal Muscle Mass Determination

We used bioelectrical impedance analysis (BIA) (InBody 770, Biospace Inc., Seoul, South Korea) to analyze the body composition of all participants. The InBody 770 device provides impedance for each segment, including the four limbs and the trunk, by performing multi-frequency measurements to estimate the appendicular skeletal muscle mass (ASM). In this study, the ASM was calculated as the sum of the lean muscle mass of the four limbs. The skeletal muscle mass index (SMI) was calculated as appendicular skeletal muscle mass (ASM) (kg) by body weight (BW) and expressed as a percentage [ASM/BW (%)] (12).

### Statistical Analysis

Continuous variables are expressed as mean ± standard deviation (SD), and categorical variables are expressed as counts and percentages. One-way ANOVA with Bonferroni correction was conducted for continuous variables and chi-square tests for categorical variables. The association of SMI with FAP and LSM was explored by Pearson correlation analysis. The independent associations of SMI with the extent of hepatic steatosis and liver fibrosis presence was performed by multinomial logistic regression analysis after adjusting for confounding factors. Significance was defined as *P* < 0.05 (two-sided). All statistical analyses were conducted using SPSS 18.0 statistical software.

## Results

### Baseline Characteristics of the Study Population

All participants were divided into the NAFLD group (*n* = 1830) and the non-NAFLD group (*n* = 1772) based on liver ultrasonography results. Characteristics of the study population at baseline are listed in Table 1. Participants with NAFLD were more likely to be male and older compared with those without NAFLD. Metabolic characteristics in those with NAFLD were considerably less favorable than in those without NAFLD. Participants with NAFLD had higher BMI, systolic blood pressure (SBP), diastolic blood pressure (DBP), FPG, HbA1c, serum uric acid and worse lipid profile such as higher triglyceride (TG), total cholesterol (TC), low-density lipoprotein cholesterol (LDL-C) and lower high-density lipoprotein cholesterol (HDL-C) (all *P* < 0.05). In addition, Participants with NAFLD also had worse liver function such as higher alanine aminotransferase (ALT) (*P* < 0.05).

**TABLE 1:** Baseline characteristics of individuals with or without NAFLD.

	Non-NAFLD (*n* = 1,772)	NAFLD (*n* = 1,830)	*P* value
Age (years)	44.32 ± 11.44	47.37 ± 10.48	< 0.01
Male (n, %)	860 (49.31)	1,467 (80.16)	< 0.01
BMI (kg/m^2^)	22.43 ± 2.34	27.06 ± 3.03	< 0.01
SBP (mmHg)	119.41 ± 16.27	129.82 ± 15.84	< 0.01
DBP (mmHg)	73.14 ± 10.45	80.81 ± 10.70	< 0.01
FPG (mmol/L)	5.07 ± 0.81	5.72 ± 1.51	< 0.01
HbA1c (%)	5.52 ± 0.67	5.83 ± 1.01	< 0.01
TC (mmol/L)	4.98 ± 0.97	5.24 ± 1.04	< 0.01
TG (mmol/L)	1.21 ± 0.75	2.11 ± 1.61	< 0.01
LDL-C (mmol/L)	2.98 ± 0.71	3.26 ± 0.74	< 0.01
HDL-C (mmol/L)	1.40 ± 0.30	1.18 ± 0.24	< 0.01
Uric acid (mmol/l)	316.02 ± 77.66	386.70 ± 85.69	< 0.01
ALT (U/L)	18.67 ± 10.14	31.91 ± 19.72	< 0.01
AST (U/L)	21.24 ± 6.64	25.27 ± 9.06	< 0.01
GGT (U/L)	24.32 ± 18.61	43.77 ± 29.40	< 0.01

*Values are presented as mean ± standard deviation. BMI, body mass index; SBP, systolic blood pressure; DBP, diastolic blood pressure; FPG, fasting plasma glucose; TC, total cholesterol; TG, triacylglyceride; LDL-C, low-density lipoprotein cholesterol; HDL-C, high-density lipoprotein cholesterol; ALT, alanine aminotransferase; AST, aspartate transaminase; GGT, gamma-glutamyl transpeptidase.*

### Skeletal Muscle Mass Index Is Associated With the Severity of Hepatic Steatosis

**FIGURE 1:**
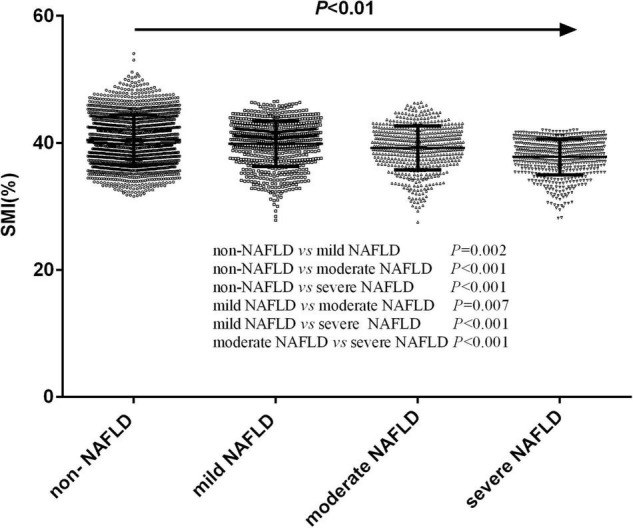
The differences of skeletal muscle mass index levels in participants with different severity of hepatic steatosis.

ALL NAFLD participants were further divided into the mild NAFLD group (*n* = 719), the moderate NAFLD group (*n* = 555) and the severe NAFLD group (*n* = 556), according to the FAP value. SMI gradually decreased in a stepwise manner as the severity of hepatic steatosis increased (40.47 ± 3.94% vs. 39.89 ± 3.57% vs. 39.22 ± 3.46% vs. 37.81 ± 2.84%, *P* < 0.01) (Figure 1). After a *post hoc* test using Bonferroni correction, there were significant differences of SMI among four groups (*P* < 0.01).

### Baseline Characteristics of NAFLD Patients by Liver Fibrosis Presence

Patients who had liver fibrosis were more frequently male, were older and had higher BMI, SBP, DBP, FPG, TG, serum uric acid, lower HDL-C and worse liver function including higher ALT than those who did not have NAFLD liver fibrosis (all *P* < 0.05) (Table 2). However, HbA1c, TC and LDL-C levels were not different (all *P* > 0.05).

**TABLE 2:** Baseline characteristics of individuals with or without liver fibrosis in NAFLD.

	Non-liver fibrosis (*n* = 1,453)	liver fibrosis (*n* = 377)	*P* value
Age (years)	47.10 ± 10.28	48.42 ± 11.15	0.04
Male (n, %)	1,149 (79.08)	318 (84.35)	0.02
BMI (kg/m^2^)	26.54 ± 2.66	29.06 ± 3.51	< 0.01
SBP (mmHg)	128.39 ± 15.30	135.33 ± 16.66	< 0.01
DBP (mmHg)	79.91 ± 10.40	84.30 ± 11.12	< 0.01
FBG (mmol/L)	5.54 ± 1.27	6.39 ± 2.06	< 0.01
HbA1c (%)	5.78 ± 0.99	6.09 ± 1.11	0.05
TC (mmol/L)	5.25 ± 1.04	5.24 ± 1.07	0.82
TG (mmol/L)	2.02 ± 1.54	2.46 ± 1.78	< 0.01
LDL-C (mmol/L)	3.26 ± 0.74	3.27 ± 0.74	0.85
HDL-C (mmol/L)	1.19 ± 0.24	1.12 ± 0.23	< 0.01
Uric acid (mmol/l)	382.98 ± 84.21	401.03 ± 89.85	< 0.01
ALT (U/L)	29.24 ± 16.47	42.21 ± 26.67	< 0.01
AST (U/L)	24.07 ± 7.69	29.90 ± 12.03	< 0.01
GGT (U/L)	41.45 ± 28.02	52.72 ± 32.75	< 0.01

*Values are presented as mean ± standard deviation. BMI, body mass index; SBP, systolic blood pressure; DBP, diastolic blood pressure; FPG, fasting plasma glucose; TC, total cholesterol; TG, triacylglyceride; LDL-C, low-density lipoprotein cholesterol; HDL-C, high-density lipoprotein cholesterol; ALT, alanine aminotransferase; AST, aspartate transaminase; GGT, gamma-glutamyl transpeptidase.*

### Skeletal Muscle Mass Index Is Associated With the Severity of Liver Fibrosis

**FIGURE 2:**
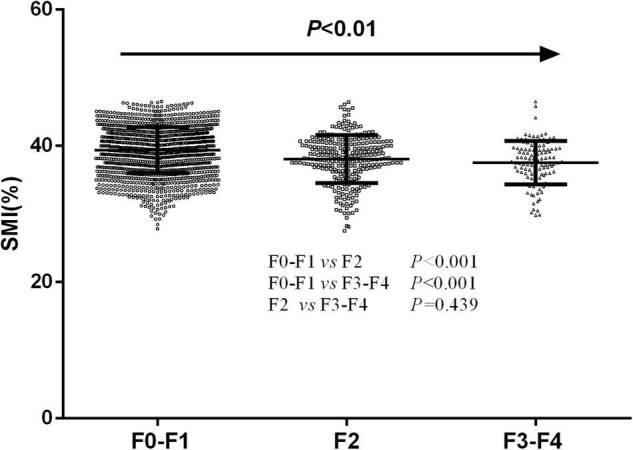
The differences of skeletal muscle mass index levels in participants with different severity of liver fibrosis.

According to LSM values, 1,453 NAFLD patients have F0-F1 fibrosis (LSM ≤ 7.3 kPa), 263 NAFLD patients had F2 fibrosis (7.3 < LSM ≤ 9.7 kPa), and 114 NAFLD patients had F3-F4 fibrosis (LSM > 9.7 kPa). Participants with F3-F4 and F2 fibrosis had significantly lower SMI than those in F0-F1 stage (37.51 ± 3.19% vs. 38.06 ± 3.51% vs. 39.36 ± 3.38%, *P* < 0.001). Those with F3-F4 fibrosis had lower SMI than those with F2 fibrosis, but this difference was not statistically significant (Figure 2).

### Logistic Regression Analysis of the Association of Skeletal Muscle Mass Index With Hepatic Steatosis and Liver Fibrosis Severity

Factors that were significantly associated with NAFLD on univariate analysis that were considered clinically significant were included. SMI was significantly correlated with mild NAFLD (*OR* = 0.755, 95% *CI* 0.674–0.846, *P* < 0.001), moderate NAFLD (*OR* = 0.676, 95% *CI* 0.598–0.763, *P* < 0.001) and severe NAFLD (*OR* = 0.538, 95%*CI* 0.468–0.619, *P* < 0.001) after adjustment for age, sex, BMI, blood pressure, lipid profile, FBG, HbA, and uric acid. In patients with NAFLD, SMI was independently associated with F2 stage (*OR* = 0.831, 95%*CI* 0.787–0.877, *P* < 0.001) and F3-F4 stage (*OR* = 0.754, 95%*CI* 0.698–0.814, *P* < 0.001) after adjustment for confounding factors such as age, sex, BMI, SBP, DBP, FPG, TG, HDL-C, and uric acid.

In addition, SMI was represented by categorical variables, and we set SMI tertiles 3 (highest SMI group) as the reference group. Logistic regression analysis showed that SMI tertiles 2 (*OR* = 1.809, 95%*CI* 1.505–2.355, *P* < 0.001) and SMI tertiles 1 (*OR* = 3.834, 95%*CI* 2.178–6.748, *P* < 0.001) were significantly correlated with NAFLD, even adjusted for confounders (Table 3). In the same way, SMI tertiles 2 (*OR* = 1.835, 95%*CI* 1.322–2.546, *P* < 0.001) and SMI tertiles 1 (*OR* = 3.714, 95%*CI* 2.597–5.312, *P* < 0.001) were independently associated with NAFLD-related liver fibrosis (Table 4).

**TABLE 3:** Multivariate analysis of the risk of non-alcoholic fatty liver disease (NAFLD) according to the tertiles of skeletal muscle mass index (SMI).

	Tertiles 3	Tertiles 2	Tertiles 1	*P* value
Model 1	[1] (reference)	2.827 (2.118–3.572)	4.139 (3.400–5.040)	< 0.001
Model 2	[1] (reference)	2.085 (1.131–2.923)	4.425 (2.629–7.447)	< 0.001
Model 3	[1] (reference)	1.809 (1.505–2.355)	3.834 (2.178–6.748)	< 0.001

*Model 1: adjustment for age, sex, BMI, systolic blood pressure (SBP) and diastolic blood pressure (DBP). Model 2: adjustment for age, sex, BMI, SBP, DBP, fasting plasma glucose (FPG) and HbA1c. Model 3: adjustment for age, sex, BMI, SBP, DBP, FPG, HbA1c, total cholesterol (TC), triacylglyceride (TG), low-density lipoprotein cholesterol (LDL-C), high-density lipoprotein cholesterol (HDL-C) and uric acid.*

**TABLE 4:** Multivariate analysis of the risk of liver fibrosis according to the tertiles of skeletal muscle mass index (SMI).

	Tertiles 3	Tertiles 2	Tertiles 1	*P* value
Model 1	[1] (reference)	2.113 (1.536–2.907)	4.275 (3.012–6.067)	< 0.001
Model 2	[1] (reference)	1.941 (1.403–2.686)	3.928 (2.755–5.601)	< 0.001
Model 3	[1] (reference)	1.835 (1.322–2.546)	3.714 (2.597–5.312)	< 0.001

*Model 1: adjustment for age, sex, BMI, systolic blood pressure (SBP) and diastolic blood pressure (DBP). Model 2: adjustment for age, sex, BMI, SBP, DBP and fasting plasma glucose (FPG). Model 3: adjustment for age, sex, BMI, SBP, DBP, FPG, triacylglyceride (TG), high-density lipoprotein cholesterol (HDL-C) and uric acid.*

### Prevalence of NAFLD and Liver Fibrosis by Skeletal Muscle Mass Index Tertiles

**FIGURE 3:**
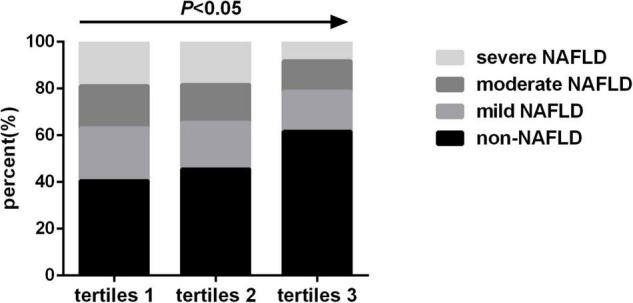
Prevalence of non-alcoholic fatty liver disease (NAFLD) by skeletal muscle mass index tertiles.

The prevalence of mild NAFLD (22.6% vs. 20.0% vs. 17.2%, *P* < 0.05), moderate NAFLD (18.0% vs. 16.2% vs. 13.0%, *P* < 0.05) and severe NAFLD (18.9% vs. 18.3% vs. 8.2%, *P* < 0.05) gradually decreased as the SMI tertiles increased, while the prevalence of non-NAFLD gradually increased as the SMI tertiles increased (40.5% vs. 45.5% vs. 61.6%, *P* < 0.05) (Figure 3).

**FIGURE 4:**
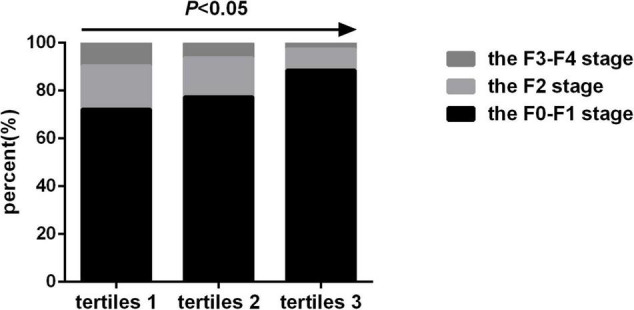
Prevalence of liver fibrosis by skeletal muscle mass index tertiles.

The prevalence of F2 stage (18.1% vs. 16.2% vs. 8.9%, *P* < 0.05) and F3-F4 stage (9.7% vs. 6.2% vs. 2.6%, *P* < 0.05) gradually decreased as the SMI tertiles increased, while the prevalence of no liver fibrosis gradually increased as the SMI tertiles increased (72.2% vs. 77.4% vs. 88.5%, *P* < 0.05) (Figure 4).

## Discussion

The present study confirms the association of SMM with hepatic steatosis and liver fibrosis determined by transient elastography in Chinese adults. Our key finding is that SMI is independently associated with the severity of hepatic steatosis and liver fibrosis related to NAFLD, after adjustment for known metabolic risk factors. When we assessed the association of SMI tertiles with NAFLD and liver fibrosis, individuals with lower muscle mass were significantly correlated with NAFLD and liver fibrosis in the multivariate analysis. These findings suggest that NAFLD and liver fibrosis are proportionally affected by the amount of skeletal muscle, even if individuals do not have overt sarcopenia.

Skeletal muscle is an insulin-responsive organ and a primary site of glucose disposal. Skeletal muscle has also been regarded as an important endocrine organ, as it secretes myokines that influence metabolic processes in muscle, liver, adipose tissue, and other organs (16, 17). During the past few years, more and more papers have reported low skeletal muscle mass (SMM) as a prevalent muscle abnormality in patients with chronic liver disease that is associated with poor prognosis (18, 19). NAFLD is the most common chronic liver disease. Low SMM reduces insulin-mediated glucose disposal and promotes insulin resistance. In line with this, the association of low SMM with NAFLD has also been reported. A large population-based 7-year longitudinal study using the HIS prediction model to diagnose NAFLD indicated that baseline highest tertiles of SMI was correlated with decreased risk of developing NAFLD and positively associated with the resolution of NAFLD if present at baseline (12). A 10-year retrospective cohort study using ultrasonography to diagnose NAFLD and a cross-sectional analysis using the fatty liver index to define NAFLD both showed that low SMI was independently associated with NAFLD, (20, 21) consistent with another cross-sectional study conducted in South Korea (22). In children, muscle mass was independently associated with both imaging and histological features of hepatic steatosis severity (23). In overweight/obese youths, low muscle mass was independently correlated with NAFLD/NASH (24). Li G et al. demonstrated that lower SMM combined with abdominal obesity is strongly associated with the severity of NAFLD, where this study used liver biopsy to evaluate the severity of NAFLD (25). Liver biopsy is recognized as the gold standard technique for diagnosing NAFLD and grading fibrosis, but owing to its invasiveness, cost, sampling heterogeneity and the risk of complications, it is not suitable for large-scale population studies. MRI-based techniques are a non-invasive modality of choice for liver fat measurement. But the MRI-based techniques are expensive and not routinely accessible. Transient elastography has been widely used as a non-invasive biomarker to assess NAFLD and liver fibrosis severity, and results compare well with liver biopsy (26). The novelty of our present study was to determine the association between SMM and the degree of hepatic steatosis in a large cohort of Chinese patients with and without NAFLD, using transient elastography. In our study, we found that SMI was negatively correlated with hepatic steatosis severity, even when adjusting for known metabolic risk factors. The proportions of participants with mild, moderate, and severe NAFLD gradually decreased as the SMI increased, whereas the proportions of individuals without NAFLD increased as SMI increased. Logistic regression analysis showed that participants in the lowest SMI tertiles had approximately 4 times risk of NAFLD compared with those in the highest SMI tertiles, independent of classic metabolic risk factors. These findings suggest that decreased SMI is an independent risk factor for NAFLD, even if overt sarcopenia is not present. The precise mechanisms underpinning the relationship between low skeletal muscle mass and development of NAFLD remain elusive, but could involve increased insulin resistance, chronic inflammation, altered secretion of the myokines myostatin and adiponectin, vitamin D deficiency and physical inactivity (27, 28).

In the past few years, the correlation between SMM and liver fibrosis in patients with NAFLD has been explored. In one systematic review and meta-analysis, the SMI in patients with NAFLD was lower than that in healthy individuals without NAFLD, and patients with sarcopenia had a greater risk of NAFLD, NASH and significant fibrosis (29). Kang MK et al. found that low SMM was independently correlated with advanced fibrosis estimated by the NAFLD fibrosis score (NFS) and the Fibrosis-4 index (FIB-4) (22). Data from the Korean National Health and Nutrition Examination Surveys 2008–2011 database (which used NFS and FIB-4 to assess liver fibrosis) indicated that sarcopenia is associated with liver fibrosis in patients with NAFLD, independent of obesity and insulin resistance (30). In individuals with T2DM with NAFLD, low SMM was independently associated with liver fibrosis estimated by Transient elastography (31). In a prospective biopsy-confirmed NAFLD cohort of 521 patients indicated that low SMM was an independent predictor of significant fibrosis, (32) consistent with another biopsy-proven NAFLD cohort study (13). Miyake et al. found that appendicular skeletal muscle mass remained associated with liver fibrosis estimated by liver biopsy after adjustment for confounding factors (33). In the present study, liver fibrosis was defined by transient elastography and similar results were obtain form our study. We found that SMI was negatively correlated with the severity of liver fibrosis, independent of metabolic factors. Moreover, the percentages of patients in F2 stage and F3-F4 stage gradually decreased as the SMI increased, while the percentages of subjects without liver fibrosis gradually increased as the SMI increased. Furthermore, logistic regression analysis showed that participants with NAFLD in the lowest SMI tertiles has a high risk of liver fibrosis than those in the highest SMI group tertiles. Therefore, in patients with NAFLD, low SMM can be considered an independent risk factor, conveying a risk of advanced fibrosis approximately 4 times that of high SMM. However, these findings need to be confirmed in a prospective, longitudinal study design.

In the same line, the role of body fat distribution in the development of NAFLD has attracted widespread attention in hepatology medicine. A study in overweight/obese adolescents showed that upper body fat distribution might play an important role in the development of NAFLD (34). Another study in adults indicated that android/gynoid ratio was significantly hepatica steatosis in both men and women. However, android/gynoid ratio was independently correlated with liver fibrosis in females, but not in males (35). In the present study, we used the InBody 770 device to determine body composition. However, the InBody 770 device cannot determine android/gynoid fat. In the future study, we will investigate the effect of body fat distribution (especially android and gynoid fat deposition patterns) on hepatic steatosis and fibrosis in Chinese population.

This study has some limitations. It is cross-sectional study conducted at a single center. A multicenter, longitudinal, prospective study are warranted to provide more definitive evidence. Second, dual-energy X-ray absorptiometry or CT imaging remain the gold standard for the measurement of SMM, but they both involve radiation exposure and are costly. We used bioelectrical impedance analysis, which is a non-invasive and simple method compared with dual X-ray absorptiometry, to assesses skeletal muscle. However, there may be a difference between fat-free mass measured by bioelectrical impedance analysis and muscle mass according to the body water content. Third, MRI-based techniques are a non-invasive modality of choice for liver fat measurement. But the expense of MRI-based techniques limits its routine clinical use. Fourth, we did not measure markers of inflammation or insulin resistance. Therefore, our study cannot provide further evidence about the possible mechanisms by which low SMM has a potential impact on the severity of hepatic steatosis and liver fibrosis. Fifth, we did not perform tests to exclude liver disease in all participants, but diagnoses were only based on self-report (e.g., viral hepatitis). Finally, because our study was based on only Chinese participants, our results may not be generalizable to other ethnic populations.

## Conclusion

In conclusion, SMM is negatively correlated with the severity of hepatic steatosis and liver fibrosis in patients with NAFLD. Intervention to increase muscle mass, especially ASM, might provide a new and important approach to prevent and manage NAFLD. Nevertheless, additional large-scale multicenter prospective longitudinal studies are needed to confirm our results.

## Data Availability Statement

The original contributions presented in the study are included in the article/Supplementary Material, further inquiries can be directed to the corresponding author.

## Ethics Statement

The studies involving human participants were reviewed and approved by Human Research Ethics Committee of the First Affiliated Hospital of Nanjing Medical University. The patients/participants provided their written informed consent to participate in this study.

## Author Contributions

WG and QZ participated in the study design. MM, XUL, XAL, PQ, JL, WZ, JW, CZ, and NX were involved in the conduct of the study and data collection. WG and XZ made contributions to data analysis and results interpretation. WG and QZ wrote and modified the manuscript and prepared tables and figures. All authors read and approved the final manuscript.

## Conflict of Interest

The authors declare that the research was conducted in the absence of any commercial or financial relationships that could be construed as a potential conflict of interest.

## Publisher’s Note

All claims expressed in this article are solely those of the authors and do not necessarily represent those of their affiliated organizations, or those of the publisher, the editors and the reviewers. Any product that may be evaluated in this article, or claim that may be made by its manufacturer, is not guaranteed or endorsed by the publisher.

## Supplementary Material

Supplementary Figure 1 The flow chart of study population.
