# Supplementary figures and images for: Association Between Skeletal Muscle Mass and Severity of Steatosis and Fibrosis in Non-alcoholic Fatty Liver Disease
Source: Front Nutr. 2022 Apr 26;9:883015. doi: 10.3389/fnut.2022.883015 (PMC9087584; doi:10.3389/fnut.2022.883015)

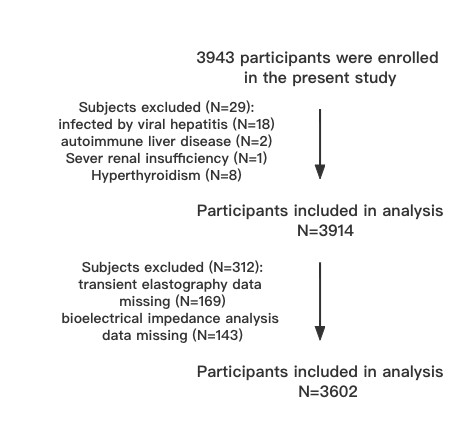

Supplement: Supplementary Figure 1 — The flow chart of study population. [file Image_1.JPEG]
